# Decomposing the effect of normal aging and Alzheimer’s disease in brain morphological changes via learned aging templates

**DOI:** 10.1038/s41598-025-96234-w

**Published:** 2025-04-07

**Authors:** Jingru Fu, Daniel Ferreira, Örjan Smedby, Rodrigo Moreno

**Affiliations:** 1 Division of Biomedical Imaging, Department of Biomedical Engineering and Health Systems, KTH Royal Institute of Technology, 14157 Stockholm, Sweden; 2 Division of Clinical Geriatrics, Centre for Alzheimer Research, Department of Neurobiology, Care Sciences, and Society, Karolinska Institute, 14186 Stockholm, Sweden; 3 Facultad de Ciencias de la Salud, Universidad Fernando Pessoa Canarias, Las Palmas, Spain; 4 Department of Radiology , Mayo Clinic, Rochester, USA

**Keywords:** Normal aging, Alzheimer’s disease, Deformation-based morphometry, Aging score, AD-specific score, Alzheimer's disease, Biomarkers

## Abstract

Alzheimer’s disease (AD) subjects usually show more profound morphological changes with time compared to cognitively normal (CN) individuals. These changes are the combination of two major biological processes: normal aging and AD pathology. Investigating normal aging and residual morphological changes separately can increase our understanding of the disease. This paper proposes two scores, the aging score (AS) and the AD-specific score (ADS), whose purpose is to measure these two components of brain atrophy independently. For this, in the first step, we estimate the atrophy due to the normal aging of CN subjects by computing the expected deformation required to match imaging templates generated at different ages. We used a state-of-the-art generative deep learning model for generating such imaging templates. In the second step, we apply deep learning-based diffeomorphic registration to align the given image of a subject with a reference imaging template. Parametrization of this deformation field is then decomposed voxel-wise into their parallel and perpendicular components with respect to the parametrization of the expected atrophy of CN individuals in one year computed in the first step. AS and ADS are the normalized scores of these two components, respectively. We evaluated these two scores on the OASIS-3 dataset with 1,014 T1-weighted MRI scans. Of these, 326 scans were from CN subjects, and 688 scans were from subjects diagnosed with AD at various stages of clinical severity, as defined by clinical dementia rating (CDR) scores. Our results reveal that AD is marked by both disease-specific brain changes and an accelerated aging process. Such changes affect brain regions differently. Moreover, the proposed scores were sensitive to detect changes in the early stages of the disease, which is promising for its potential future use in clinical studies. Our code is freely available at https://github.com/Fjr9516/DBM_with_DL.

## Introduction

Alzheimer’s disease (AD) is a neurodegenerative disorder characterized by progressive cognitive decline and widespread brain atrophy^1^. One of the greatest challenges in understanding AD is that the morphological changes observed in brain MRI scans are not solely related to the disease. Even in the asymptomatic and prodromal stages, these changes are also influenced by normal aging and patient-specific factors such as clinical history^2^. Thus, a relevant question to answer is to which extent AD can be seen as a disease that accelerates the normal aging process.

In recent years, the development of brain age prediction models has gained significant attention. These models, trained on Magnetic Resonance Imaging (MRI) data from cognitively normal (CN) individuals, estimate a subject’s brain age relative to their chronological age^3–7^. Convolutional neural networks (CNNs) have proven effective in predicting brain age using structural MRIs^5,8,9^. A negative difference between predicted brain age and chronological age is often regarded as a biomarker of neurodegenerative diseases, including AD^10^. The main assumption of this methodology is that AD can be modeled as an accelerated aging process. One issue of *global* brain age models is that they lack interpretability when it comes to understanding localized morphological changes in specific brain regions, such as the hippocampus, which are disproportionately affected by AD^11^.

To capture *regional* morphological changes, deformation-based morphometry (DBM) has been widely used. DBM characterizes these changes through non-linear registration, which estimates spatial transformations (i.e., deformations) between images^12^. One big advantage of DBM compared to brain age is that it does not constrain AD to be an accelerated aging process. This makes it possible to disentangle the accelerated normal aging process from other factors in AD, as is done in this and previous studies^2,13^. In a typical DBM workflow, morphological changes are compared across subjects by spatially normalizing brain images to a study-specific template. However, creating study-specific templates and aligning individual trajectories can be computationally expensive due to the high dimensionality of brain MRI data^14–16^. This process often requires extensive non-linear registration and can involve resource-intensive algorithms like parallel transport.

**Fig. 1:**
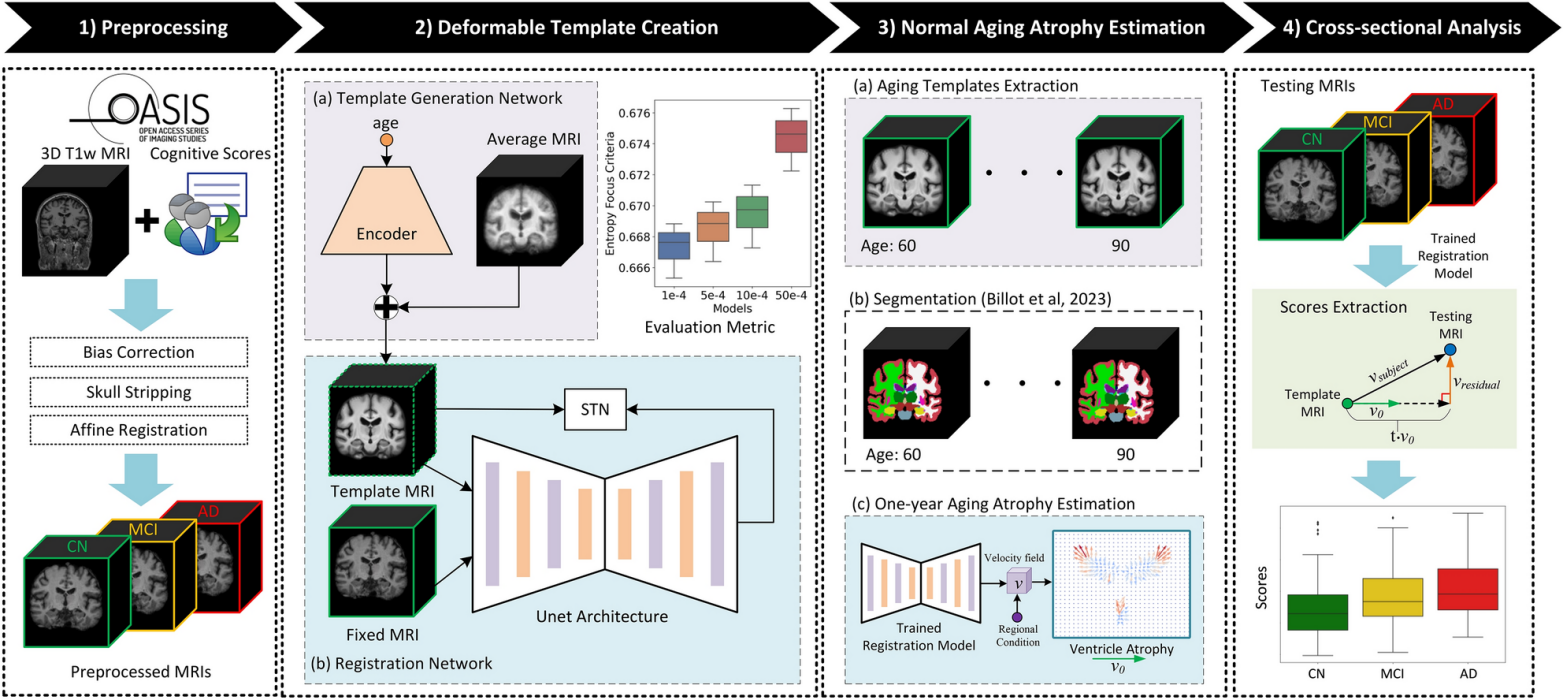
Schematic of the proposed pipeline. (**1**) Preprocessing: OASIS-3 dataset was employed in this study, 3D T1w MRI scans and the corresponding subject-level clinical diagnosis were collected, scans were processed and partitioned into cognitively normal (CN), mild cognitive impairment (MCI), and AD groups. Note that the three groups illustrated in this figure represent a simplified progression of AD; (**2**) A deep learning-based template creation method was employed to generate age-specific CN templates. (**3**) CN templates from the year range of interest were generated and segmented. Deep learning-based diffeomorphic registration was used to estimate the stationary velocity field (SVF) that describes the expected one-year healthy atrophy. Segmentation masks were provided to obtain regional estimations of normal aging atrophy. (**4**) Deformation field between a given image and a reference template was decomposed into its aging and AD-specific components by comparing each vector with the one-year healthy atrophy vector field. These components were used to estimate aging score (AS) and AD-specific score (ADS). These scores were used for group comparisons. STN: Spatial transformer network.

Given these challenges, we propose a novel deep-learning-based DBM framework that combines deformable template creation and registration in an efficient manner. Our approach bypasses the need for longitudinal data by directly learning templates from cross-sectional data using a generative model^17^. Unlike traditional methods that require computationally intensive steps like subject-specific template creation and parallel transport, our framework leverages deep learning to streamline the DBM workflow. By utilizing stationary velocity fields (SVFs) learned from CN subjects, we model one-year atrophy patterns and disentangle normal aging from AD-specific morphological changes. This approach allows us to introduce two orthogonal scores—following assumptions presented in previous work^2^, the aging score (AS) and the AD-specific score (ADS), providing a finer understanding of the progression of brain atrophy in AD. Our method differs from the previous approach^2^ in several key aspects: i) Cross-sectional data usage: Unlike the original method, which relies on longitudinal data to build the normal aging model, our framework uses a generative model that requires only cross-sectional data. This expands its applicability to cases where longitudinal data is scarce. ii) Introduction of an additional biomarker (ADS): In addition to the aging score (AS)—which was introduced in the original work as *morphological age shift*—our method introduces ADS, which quantifies the severity of disease-related morphological changes. This complementary biomarker provides additional insights and enhances the potential for early AD detection. iii) Preclinical validation: We validated our approach using a dataset with a larger proportion of preclinical data, enabling a more comprehensive assessment of its utility for early detection of AD. iv) Learning-based registration: By leveraging learning-based registration, our method significantly accelerates the DBM workflow. To the best of our knowledge, this is the first DBM framework for AD studies to employ a learning-based approach. To further contribute to the field, we provide a step-by-step practice guide for extracting these two scores from any MRI scan, which is available at https://github.com/Fjr9516/DBM_with_DL (Fig. 1).

The methodology was developed and evaluated using the OASIS-3 dataset, which includes 2,366 T1-weighted (T1w) MRI scans. Of these, 1,678 scans were from CN subjects, and 688 scans were from individuals clinically diagnosed with AD at various stages of clinical severity, as defined by Clinical Dementia Rating (CDR) scores. By correlating the proposed scores with CDR scores, we show that our framework successfully separates normal aging from disease-specific atrophy. This allows us to provide new insights into AD progression and offers a more precise tool for early diagnosis and therapeutic interventions.

## Materials and methods

**Table 1:** Summary of OASIS-3 dataset.

	$\#$ Subjects	$\#$ T1w scans
Collected	1316	2681
Excluded (*Quarantined* QC/undefined CDR)	1	3
Remained (CN/AD dataset/non-AD dementia)	1315 (739/419/157)	2678 (1678/688/312)
Included in this study (CN/AD dataset) CDR for AD progression dataset at MRI (0/0.5/1/2)	1158 (739/419) (143/246/94/4)	2366 (1678/688) (276/307/101/4)

The dataset includes subjects with varying CDR scores. Note that the total number of CDR-rated subjects may not equal the number of AD subjects because some participants have scans across multiple CDR stages. Additionally, some individuals diagnosed with AD may have initially presented with a CDR of 0, as OASIS-3 is a retrospective dataset, and subjects may have converted to AD at a later stage.

### Dataset

The Open Access Series of Imaging Studies - version 3 (OASIS-3) dataset^18^ was used to train the deformable template generation model and conduct the evaluations. OASIS-3 is unique in that it focused on a pre-clinical cohort and followed their longitudinal progress. OASIS-3 is a compilation of data for 1,378 participants, including 755 cognitively normal adults and 622 individuals at various stages of cognitive decline ranging in age from 42-95 years. It contains over 2,000 magnetic resonance (MR) sessions and includes T1w MR scans, among other sequences. Many of the MR sessions are accompanied by volumetric segmentation files produced through FreeSurfer processing (https://surfer.nmr.mgh.harvard.edu/). OASIS-3 is a longitudinal multimodal neuroimaging, clinical, cognitive, and biomarker dataset for normal aging and AD (https://www.oasis-brains.org/##about). In this study, we used T1w scans. It is important to note that the numbers presented here represent the raw dataset before applying the inclusion criteria outlined in the *Data Preparation* section. The final dataset, filtered and curated for this study, is described in Table 1.

### Data preparation

We collected the FreeSurfer processed OASIS-3 dataset released in July 2022. A summary of this collection is shown in Table 1, in which the number of subjects and scans are counted separately. There were 2,681 successfully collected scans, in which two scans failed FreeSurfer Quality Control (QC) procedure (i.e., marked as *Quarantined*) and one scan failed to define the CDR score, which is an inclusion variable in our study. Therefore, these scans were discarded for the study. We filtered the remaining scans according to the diagnoses provided in OASIS-3 and further removed non-AD dementia types to keep the focus on AD progression in our study. In this cohort, the clinical stage was defined by CDR following standards, as follows: a CDR of 0 corresponds to normal cognitive function, CDR = 0.5 indicates very mild cognitive impairment, CDR = 1 indicates mild dementia, CDR = 2 indicates moderate dementia, and CDR = 3 indicates severe dementia. According to OASIS-3’s acquisition protocol, participants who reached CDR = 2 were no longer eligible for in-person assessments. For this reason, subjects with CDR = 2 are scarce in this dataset and CDR = 3 are non-existent. After our selection steps and curation of data, 2,366 scans from 1,158 subjects were included in the study. We defined CN as subjects with CDR = 0 at all visits, and these were the scans used for the dataset of normal aging in our study. 1,678 scans from 739 subjects complied with this requirement. In turn, the AD cohort was composed of individuals who progressed to clinical AD dementia at some point during follow-up visits but could have a CDR of 0 or > 0 at the baseline. Notice that although some scans of the AD group can have CDR = 0, they are not mixed with the CN group since these images might already show some signs of AD progression that are not yet clinically signaled by the CDR test. Thus, we used 688 images from 419 subjects for the AD group with CDR scores from 0 to 2. Table 1 shows the breakdown of CDR scores in the AD progression dataset.

**Fig. 2:**
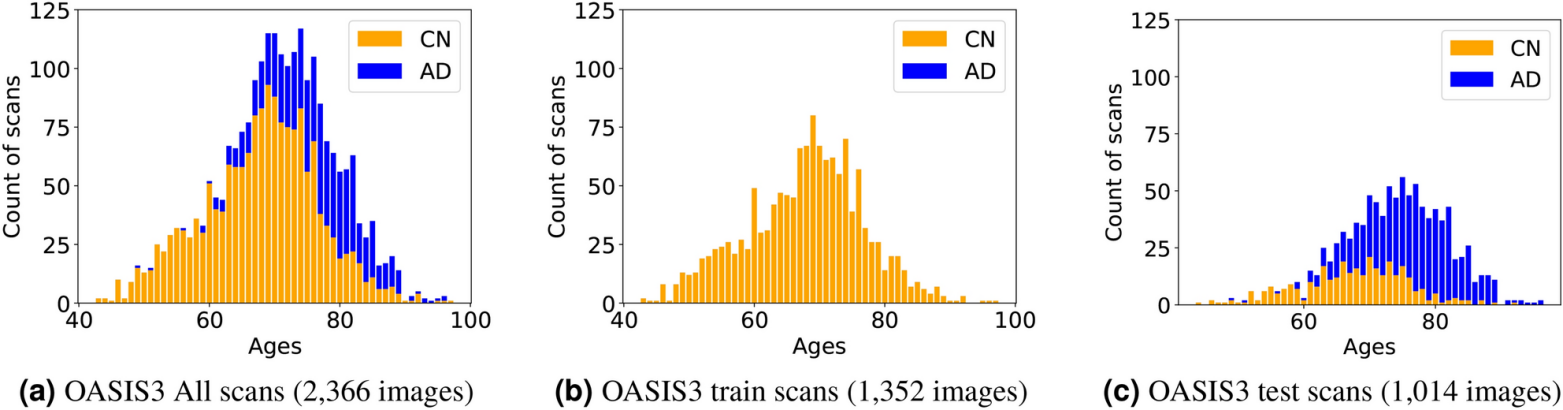
Histograms of sample size versus scan age for selected OASIS-3 scans and the training and test sets constructed in this study. The CN cohort (1678 scans) was split into 1352 scans for training (panel (b)) and 326 scans for testing (panel (c)), ensuring a similar age distribution across both sets. The AD cohort (688 scans) was included only in the test set (panel (c)). Note that only the CN cohort was used for training, as shown in panel (b).

Our approach includes the use of templates for normal aging brains. For this, it is essential to ensure sufficient data coverage across different age groups. To achieve this, we constructed the training and test sets in proportion to the age distribution of the CN datasets. Approximately 80$\%$ of the CN data was allocated to the training set, while the remaining 20$\%$ was assigned to the test set. Only the test set was used for the results reported in Sect. 3. Figure 2 illustrates histograms that depict the partitioning of the dataset, with 1,352 images assigned to the training set and 326 images assigned to the test set. In addition, the AD dataset, which contains 688 images, is used only for testing.

### Neuroimaging processing

We followed the protocol as previously outlined^17^ to prepare the suitable format data to train the deformable template generation model. Specifically, FreeSurfer processed OASIS-3 T1w data were obtained (i.e., *norm.mgz*). FreeSurfer performs skull-stripping and bias field correction according to the FreeSurfer process flow (https://surfer.nmr.mgh.harvard.edu/fswiki/ReconAllDevTable). Then, affine registration was applied to the image using the FreeSurfer *mri_vol2vol* command, utilizing Talairach space encoded in *talairach.xfm* to Montreal Neurological Institute (MNI) 305 space. Then the segmentation masks for each image can be obtained by SynthSeg^19^, which is the state-of-the-art for brain image segmentation. We rescaled the intensity of images to the range [0,1]. Finally, we cropped the input scan in the size of [208, 176, 160] to speed up training for this 3D problem and reduce the computational demands.

### Generation of imaging templates with deep learning

The goal of template generation is to estimate an image that can represent a specific population. For this, deformable image registration is employed to create such templates in order to make them unbiased and barycentric by minimizing the average geodesic distance to each individual subject in the population^17,20–23^. Traditional template creation methods are often time-consuming, particularly for obtaining high-resolution anatomical templates^21,22^. However, recent advancements in deep learning have alleviated the computational demands during the template estimation process^17,24^.

In this work, we selected AtlasGAN^17^, which is known for preserving distinguishable anatomical boundaries. AtlasGAN enables the estimation of realistic anatomy by utilizing generative adversarial learning to produce optimal conditional templates. The “Deformable Template Creation” panel of Fig. 1 shows a scheme of the method, which consists of three sub-networks: template generation, registration and discriminator sub-networks. The first two sub-networks are responsible for generating the conditional template and deforming it to match a fixed image, which is then evaluated by the discriminator sub-network. The discriminator sub-network is omitted from Fig. 1 for simplicity. For more detailed information, please refer to the original paper^17^.

Evaluation Metric: To quantify the sharpness of the template, we used the entropy focus criterion (EFC)^25^, which has been extensively used in previous studies^17,21,26–28^. Specifically, the EFC is defined as:

1 $$\begin{aligned} EFC = - \sum _{i=1}^{N} \frac{B_{i}}{B_{max}} \ln \left[ \frac{B_{i}}{B_{max}}\right] , \end{aligned}$$

with *N* being the number of image voxels, and $B_{i}$ the value of the i-th image voxel. The largest possible voxel brightness would be obtained if all the energy in the image were in one voxel, given by:

2 $$\begin{aligned} B_{max} = \sqrt{\sum _{i=1}^{N}B_{i}^2} \end{aligned}$$

In this scheme, $EFC=0$ is achieved when all the image energy is located in one voxel, while the maximum entropy is achieved when the image is uniformly gray. In other words, the sharper the image, the smaller the EFC value. We also applied a mask to the EFC to remove the background and normalized the EFC value to the range of [0, 1].

### Normal aging atrophy estimation through diffeomorphic registration

As mentioned, brain atrophy can be caused by both aging and disease, so it is necessary to disentangle these two processes^2,13,29^. For this, we first need to create a model of atrophy due to normal aging. In particular, we estimate normal aging atrophy in three steps: 1) normal aging template estimation, 2) template segmentation, and 3) one-year normal aging atrophy estimation, as illustrated in the panel “Normal Aging Atrophy Estimation” of Fig. 1.

First, sharp and age-specific CN templates were extracted with the template generation block described in the previous subsection. These templates serve as barycentric representations at different ages. Next, segmentation masks for each template are obtained using SynthSeg^19^. We used this method since it has been reported to be faster and more accurate than traditional methods, such as FreeSurfer. Finally, we used diffeomorphic registration to represent morphological changes that are expected to happen in one year in a healthy subject. For this, we re-used the diffeomorphic registration block from AtlasGAN, which we trained before. Such a block is a VoxelMorph-like network^30^. Diffeomorphic registration aims to estimate a smooth, differentiable, and invertible transformation to avoid tissue folding in the registered biological image pairs (e.g., tissues should not fold or disappear with age^31^). The transformation, referred here to as the diffeomorphic deformation field, is obtained by solving an ordinary differential equation (ODE) parameterized by a SVF $\varvec{v}$:

3 $$\begin{aligned} \frac{d\varvec{\phi }^{(t)}}{d t} = \varvec{v}(\varvec{\phi }^{(t)}) \end{aligned}$$

where $\varvec{\phi }^{(0)}$ is initialized with an identity transform. The final deformation field $\varvec{\phi }^{(1)}$ is obtained by integrating over unit time as follows:

4 $$\begin{aligned} \varvec{\phi }=\varvec{\phi }^{(1)} = \displaystyle \int _0^1 \varvec{v}(\varvec{\phi }^{(t)}) {d t}. \end{aligned}$$

Intuitively, an SVF encodes the anatomical changes between the registration pair. Thus, the one-year normal aging atrophy can be estimated by dividing the SVF that represents the registration between two CN templates by their age gap. In this study, we used the SVF between the 60-year- and 90-year-old CN templates divided by 30 as the one-year normal aging atrophy map. We refer to this SVF as $\varvec{v}_0$. We assume linear atrophy due to normal aging atrophy in line with previous studies^32,33^. We chose this age range because it provides a richer representation of data in the training set, capturing a wider range of normal aging patterns. Additionally, using templates that are further apart in age makes the estimated changes less sensitive to registration errors, improving the robustness of the aging atrophy estimation.

Various studies have shown that different brain structures age differently^32,34,35^. Thus, it is relevant to perform regional analyses by using segmentation masks to derive regional normal aging atrophy maps.

### Extraction of aging and AD-specific scores

The next step is to use normal aging atrophy, estimated as described in the previous section, to disentangle aging and AD atrophy for a specific subject. Such a separation is summarized in the newly proposed AS and ADS.

First, we build upon the assumption by^2^ that states that normal aging and disease-specific components of a specific subject can be disentangled by orthogonally projecting the subject-to-template SVF $\varvec{v}_{subject}$ onto the one-year normal aging SVF $\varvec{v}_{0}$ (see the panel “Cross-sectional Analysis” of Fig. 1). Then, the AS and ADS are computed as follows:

5 $$\begin{aligned} AS = \frac{<\varvec{v}_{subject}, \varvec{v}_{0}>}{||\varvec{v}_{0}||^2}, \end{aligned}$$

6 $$\begin{aligned} ADS = ||\varvec{v}_{subject} - AS \cdot \varvec{v}_0||, \end{aligned}$$

where $<\cdot ,\cdot>$ is the inner product of vectors. These two scores are computed voxel-wise. AS measures the alignment of specific aging of the subject with the aging process in a CN population.

Negative and positive values of AS indicate that the subject is aging at a lower or higher pace than the CN population, respectively. Notice that the AS only captures variability within normal aging. That means that a specific subject might look very old (or very young) with respect to a population and still be cognitively normal. In turn, ADS aims to capture patterns that are not present in the CN population. Thus, ADS could potentially be more discriminative of patients and controls than AS. Notably, because AS is defined relative to a one-year normal aging velocity field, it is directly proportional to chronological age changes. In contrast, ADS represents the magnitude of the residual velocity field, making it independent of age-proportional scaling and more sensitive to disease-related deviations. This difference in scale explains the varying magnitudes observed for AS and ADS in Figs. 6 and 9.

The clinical onset of AD is usually around 65 years of age, but the disease process is known to start a decade or more before the clinical onset of dementia^36^. A 60-year-old reference healthy template was used in this study to perform the subject-to-template diffeomorphic registration to estimate $\varvec{v}_{subject}$. As in the case of $\varvec{v}_0$, we used the trained diffeomorphic block from AtlasGAN for performing the registration. Although the selection of this template is arbitrary, we chose 60 years old since 60 is not too far from the typical onset of AD, and it is not too close to get too small SVFs. Notice that we use a single $\varvec{v}_0$ for the whole population. In this way, we can capture the central tendency of the aging process across a population, providing a reference point for distinguishing normal aging from disease-related changes.

**Fig. 3:**
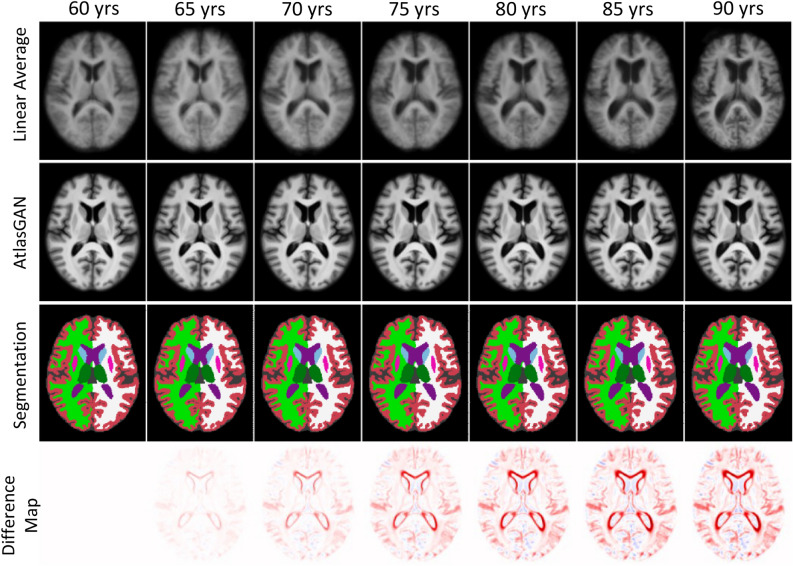
Examples of the learned templates with the corresponding segmentations at the 80th axial slice. *First row*: The linear average images for different ages; *Second row*: The templates learned with AtlasGAN for different ages; *Third row*: The segmentation masks obtained with SynthSeg^19^. The segmentations are shown using the same color scheme used in Freeview (https://surfer.nmr.mgh.harvard.edu/fswiki/FsTutorial/AnatomicalROI/FreeSurferColorLUT); *Fourth row*: residual maps obtained by subtracting the 60-year-old template from the corresponding template of every column for AtlasGAN. These images are available in 3D in our GitHub repository for better visualization.

### Outlier rejection

The calculation of the scores involves averaging voxel-by-voxel values, making the process sensitive to outliers that can arise due to inter-subject variability and registration inaccuracies. Moreover, the voxel-wise scores are influenced by the magnitude of the unit-year healthy atrophy SVF $\varvec{v}_{0}$. When $\varvec{v}_{0}$ is small, even slight discrepancies can result in disproportionately large score values, leading to potential distortions in the overall score. To mitigate this issue, we implemented a robust thresholding strategy based on the norm of $\varvec{v}_{0}$, rejecting outliers that could skew the final score calculations. Our experiments (see Fig. 5) show that the thresholding method effectively reduces the impact of outliers, leading to more accurate and reliable score estimations. Detailed results and analysis are presented in subsequent sections.

## Results

### Creation of sharp longitudinal templates with AtlasGAN

**Fig. 4:**
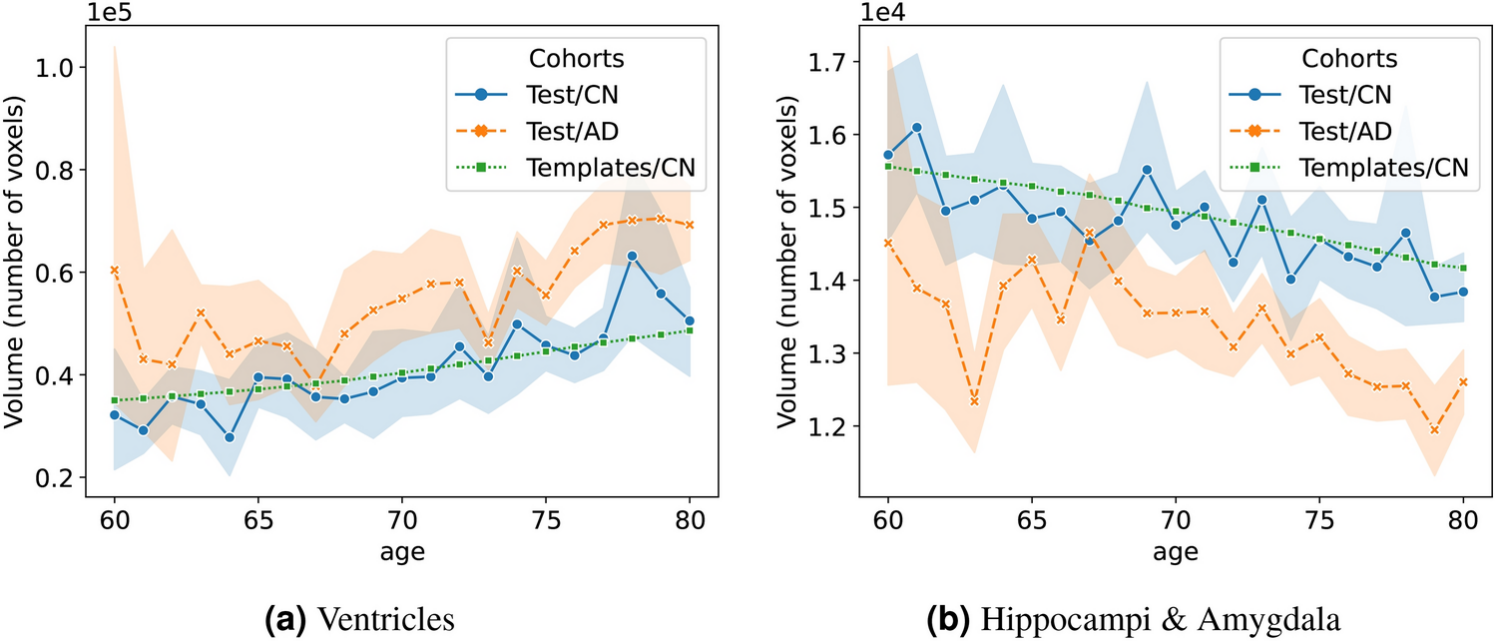
(**a**) Ventricles volumetric trend of learned templates and the real trends for AD and CN cohorts in the test sets, respectively. (**b**) Hippocampi & Amygdala trends of learned templates and the real trends for AD and CN cohorts in test sets, respectively. The standard deviations of AD and CN testing subjects are shown in light orange and blue, respectively.

Imaging templates with distinguishable boundaries among brain regions are advantageous for increasing the accuracy of downstream tasks such as registration or segmentation^17^. To quantify the sharpness of the images, we applied the EFC to the learned templates^25^. We extracted healthy templates at each integer age from 60 to 90 years old since this range is the most common in the AD population overall and is well represented in the dataset. The model trained with $\lambda _{gp}=1e^{-4}$ showed the smallest EFC (i.e., sharpest) templates, so it was chosen to be used in the subsequent analyses. Figure 3 visually compares the learned templates with linear averages. As shown, the learned templates preserve better the edges and boundaries of the different structures. This property is crucial to increase the accuracy of the downstream registration steps.

**Fig. 5:**
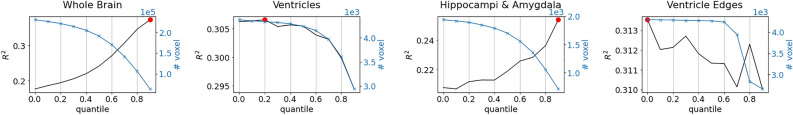
$R^2$ (in black) and the number of preserved voxels after thresholding $\varvec{|}|v_0||$ (in blue) with different quantiles for each region. The highest $R^2$ is indicated by a red dot in the figure.

### Estimation of normal aging atrophy trends using learned templates

Figure 3 also shows that the learned templates might be able to capture atrophy patterns due to normal aging (see the difference maps in the figure). To confirm this observation, we compared changes with age in segmentation volumes in real data and the learned templates for specific brain regions. For this, we ran SynthSeg both on the templates and the CN and AD real test sets. Figure 4 shows the volumetric changes with age for the ventricles (left) and hippocampi & amygdala (right) (Segmentation labels: [4, 14, 15, 43] for ventricles and [17, 53, 18, 54] for Hippocampi & Amygdala from SynthSeg). As shown, changes in regional volumes with age in the templates (depicted in green) are close to the mean of the changes in CN (depicted with a solid blue line). This means that the learned templates can capture the atrophy trends in the normal population. Thus, the templates can be used as surrogates of atrophy due to normal aging. Notice that Fig. 4 also shows that the atrophy in AD (in orange) has a different trend compared to CN. It is important to remark that the number of testing AD subjects below 65 years old is relatively limited. Thus, the estimations for AD in Fig. 4 are less reliable below that age.

### Aging and AD-specific scores applied to cognitively normal subjects

**Fig. 6:**
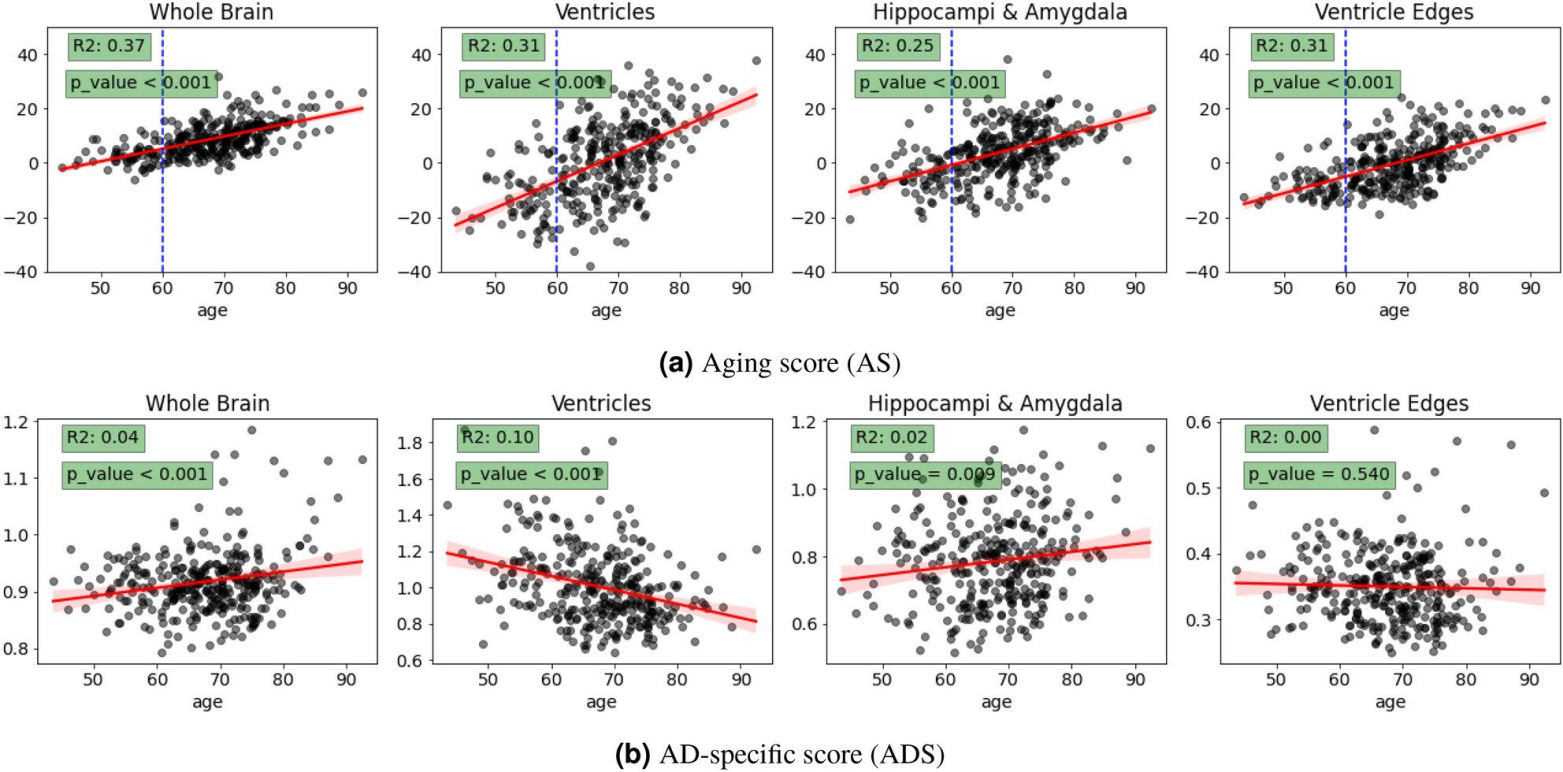
(**a**) The relationship between AS and age per brain region on the CN test set. The reference age is indicated by the blue dashed line. (**b**) The relationship between ADS and age per brain region on the CN test set. The fitted lines, shown in red, are accompanied by 95$\%$ confidence interval (CI) shadow regions in both AS and ADS plots.

As mentioned, we estimated $\varvec{v}_{0}$ using the templates at 60 and 90 years old. We actually tested SVFs created from templates with a smaller age difference, but they were less consistent. This could be attributed to the fact that SVFs can become less reliable when the morphological changes in the brain are more subtle.

Since the AS can be affected by outliers, we used different quantile values ranging from 0 to 0.9 with an interval of 0.1 to threshold $\varvec{v}_0$. We aimed to find the quantile value that maximizes the $R^2$ value, which indicates the goodness of fit between the AS and age for the CN group. The test set consisted of scans from subjects with consistent CDR scores of 0 over scanning sessions. As mentioned before, a mask was applied in every calculation to mitigate the influence of the background.

**Fig. 7:**
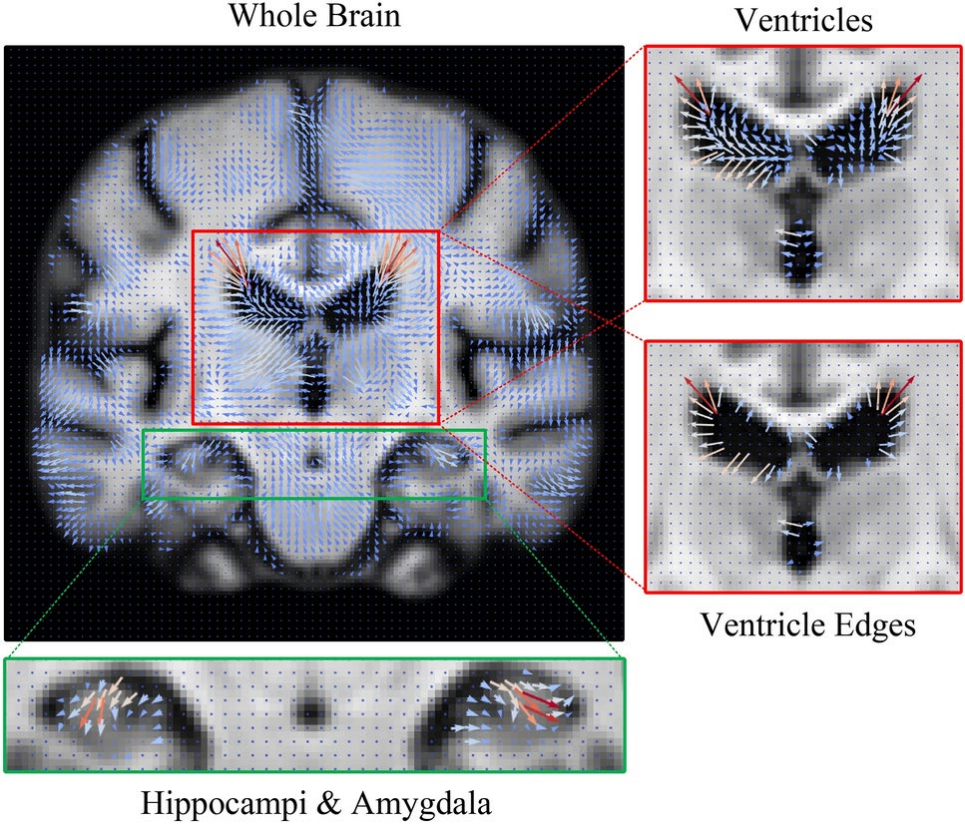
Visualization of 1-year stationary velocity field (SVF, $\varvec{v}_0$) overlaid on the learned template at age 90 in the coronal plane. To enhance clarity, the vector field resolution has been downsampled by a factor of 2, and the vectors have been rescaled. Four brain regions are presented: the whole brain, ventricles, hippocampi & amygdala, and the ventricle edge map. The ventricle edge map represents a region near the ventricular boundaries, defined as the difference between the ventricular segmentations of the 90-year-old and 60-year-old templates. Note that in the whole-brain map, edge artifacts outside the brain are filtered using a segmentation map to reduce bias.

We tested AS on four different brain regions: the whole brain, ventricles, hippocampi & amygdala, and the ventricle edge map. The ventricle edge map represents a region close to the edges of the ventricles and is defined as the difference between the ventricles segmentation of the 90-year-old and 60-year-old templates (see example in Fig. 7). The results are shown in Fig. 5. As depicted, different quantile values yielded the best fit for different brain regions. The number of preserved voxels at each quantile value is shown on the right y-axis.

The optimal fitted results for AS are shown in Fig. 6a, where the x-axis represents chronological age and the y-axis represents the AS. When the morphological age aligns with the chronological age, the fitted line is expected to intersect at 0 for age 60 and exhibit linear changes with increasing age^32,33^. As illustrated in the figure, the ventricle region provides the best fit (evidenced by a slope closer to 1), with an $R^2$ value around 0.31, indicating that ventricle enlargement closely follows chronological aging. This supports the idea that ventricle enlargement is a well-known marker of normal aging, effectively captured by the AS model. The other three regions, while showing a weaker fit with $R^2$ values between 0.25 and 0.37, still align with the expected trend of positive changes in cognitively normal individuals, reflecting the region-specific nature of aging.

For ADS, the fits are much weaker, with $R^2$ values ranging from 0.00 to 0.10 across all four regions, as shown in Fig. 6b. This suggests that ADS does not have a strong linear relationship with age in cognitively normal individuals. The low $R^2$ values for ADS indicate that, after accounting for normal aging effects (as captured by AS), the residual variance attributed to AD-specific atrophy is minimal in this population. This is expected in cognitively normal individuals, where AD-specific atrophy is likely negligible or undetectable. Notice that the reported trends for ADS are not relevant due to their low $R^2$ (e.g., the trend of ADS in the ventricles is negative but with an of just $R^2=0.1$).

Overall, the contrasting $R^2$ values for AS and ADS highlight the difference between normal aging and AD-specific processes. While the AS model captures aging-related changes with moderate accuracy, especially in the ventricles, the ADS model shows little age dependence in this healthy control group, as expected.

### Aging and AD-specific scores applied to assess the progression of AD

**Fig. 8:**
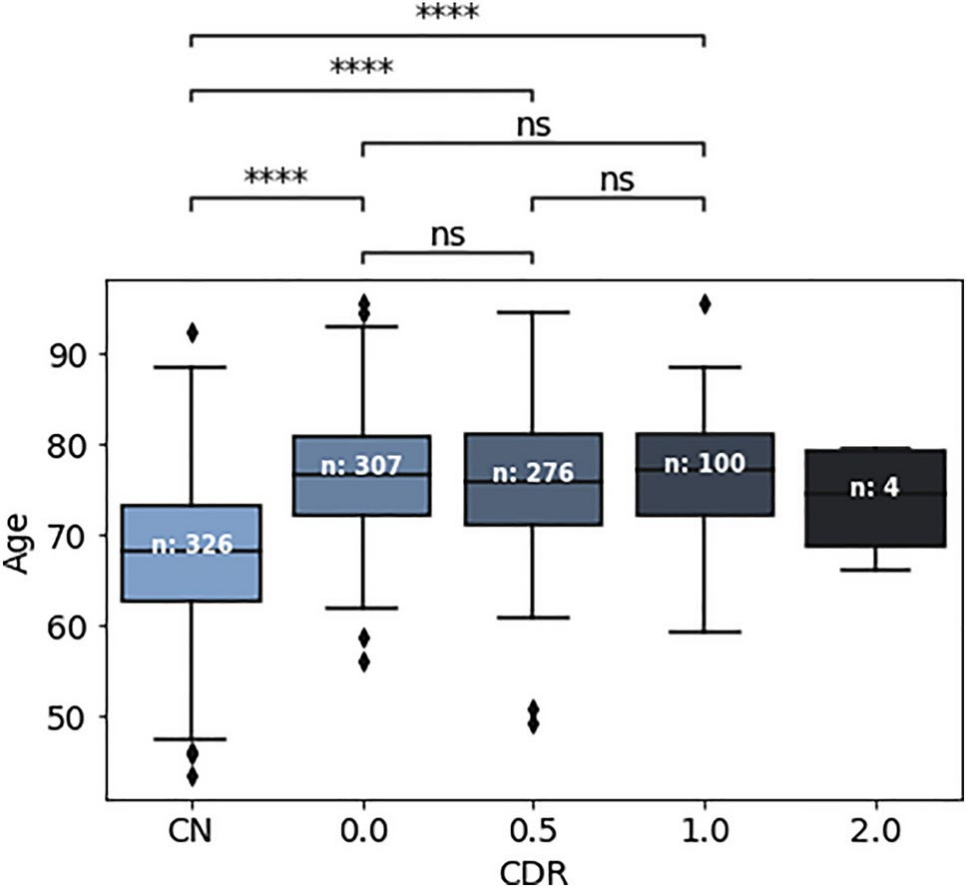
Chronological age distributions of the different cohorts (number of observations is shown in the middle of each boxplot as *n*). No significant difference was observed among the AD subgroups regarding chronological ages.

**Fig. 9:**
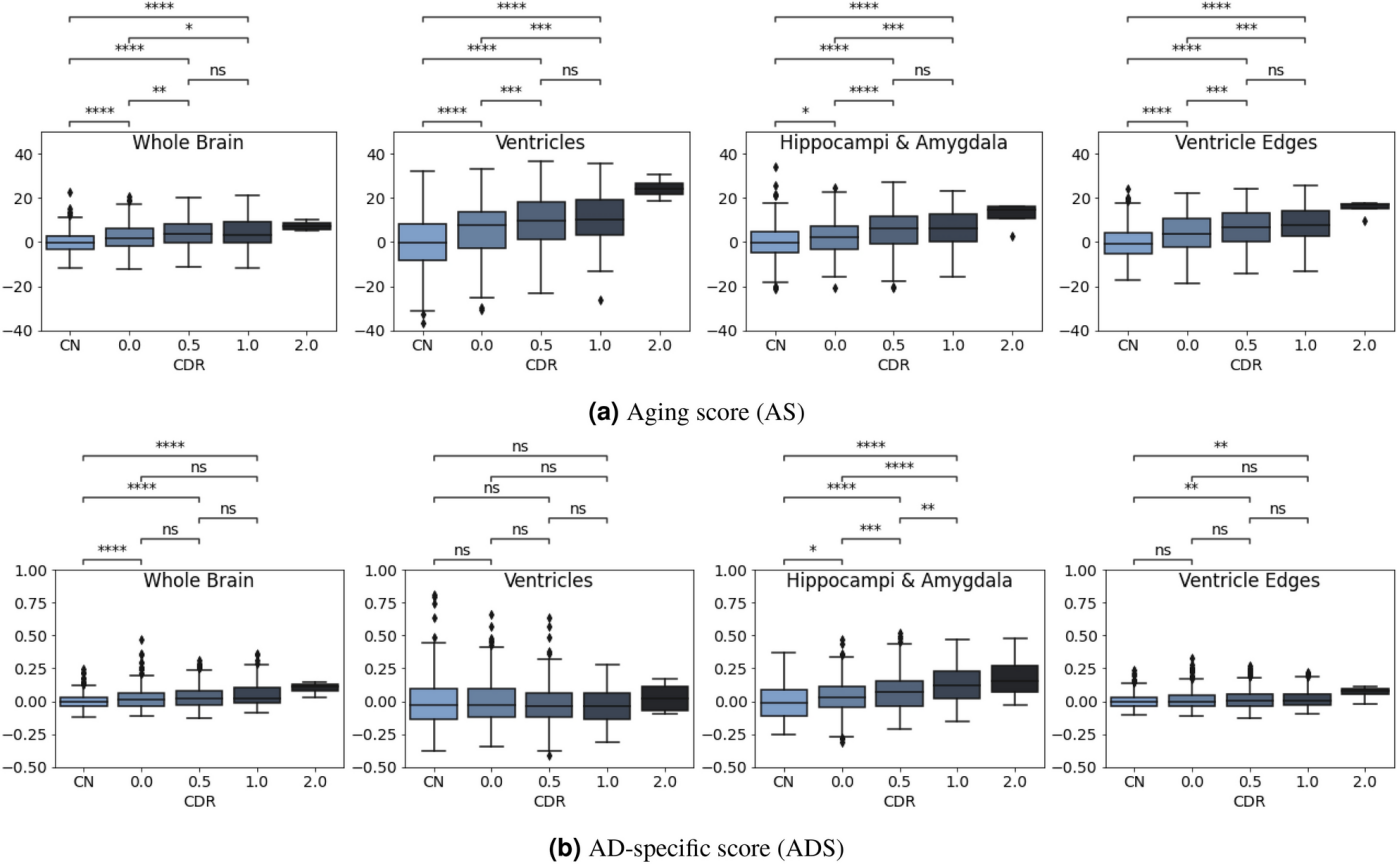
Distributions of the aging and AD-specific scores for different disease stages defined by CDR and cognitively normal subjects (CN).

To evaluate the proposed scores’ ability to differentiate the progression of AD, the AD group was divided into four subgroups (i.e., CDR = 0, CDR = 0.5, CDR = 1, and CDR = 2) according to CDR scores at the time of MRI scanning. As mentioned before, all these subjects progressed to clinical AD dementia at follow-up evaluations. Independent t-tests were performed on each pair of distributions, and the Bonferroni method was used for multiple testing corrections (The p-value annotation legends in the Figs. 8 and 9 are as follows: ns: p > 0.05; *: 0.01 < p $\le$ 0.05; **: 0.001 < p $\le$ 0.01; ***: 0.0001 < p $\le$ 0.001; ****: p $\le$ 0.0001). In the course of this experiment, a notable outlier emerged within the AD subgroup with CDR = 1. Upon visual inspection, it was determined that this outlier had failed to undergo proper affine registration in the FreeSurfer workflow. As a result, it was excluded from subsequent experiments.

Given the significant correlation of AS with chronological age, as illustrated in Fig. 6a, we examined the distributions of chronological age among the CN cohort and the subgroups of AD individuals stratified into clinical stages based on CDR, as shown in Fig. 8. The results revealed no significant age differences between any pair of AD subgroups, while a noteworthy age-related difference was observed when comparing the CN group to the AD groups.

**Table 2:** Cohen’s d effect size measure for the statistical tests of Fig. 9.

Regions		Pairs					
		(CN, 0)	(CN, 0.5)	(CN, 1)	(0, 0.5)	(0, 1)	(0.5, 1)
Whole Brain	AS	*-0.40*	-0.70	-0.82	-0.27	*-0.35*	-0.07
	ADS	*-0.36*	*-0.48*	-0.75	-0.10	-0.29	-0.20
Ventricles	AS	*-0.43*	-0.74	**-0.90**	-0.32	*-0.47*	-0.15
	ADS	-0.01	0.13	0.16	0.13	0.17	0.05
Hippocampi & Amygdala	AS	-0.22	*-0.61*	-0.68	*-0.41*	*-0.49*	-0.06
	ADS	-0.24	*-0.56*	**-0.95**	-0.32	-0.71	*-0.37*
Ventricle Edges	AS	*-0.47*	-0.80	**-1.04**	-0.31	*-0.50*	-0.19
	ADS	-0.15	-0.26	*-0.38*	-0.09	-0.18	-0.09

The pairs match cognitively normal subjects (CN) and AD subjects with different CDR scores (0, 0.5, or 1). Note that the negative Cohen’s d indicates that the mean of the first group is lower than that of the second group, while the value is related to the degree of effect size. Medium effect size (in italics): [0.35, 0.65), Large effect size (underlined): [0.65, 0.9), Very large effect size (in bold): > 0.9.

To remove the influence of age in the AS and ADS, a normalization procedure was employed. This involved the utilization of Analysis of Covariance (ANCOVA) for each score across levels of categorical disease groups, while statistically controlling for the age effect. The resulting adjusted scores were then harmonized on the same scales by subtracting the mean of the CN group for all four observed brain regions to facilitate more meaningful comparisons. As shown in Fig. 9a, there are increasing trends in the AS with respect to increasing disease stages defined by CDR scores in all four regions. This finding supports the assumption that AD is a factor for accelerated normal aging and that this acceleration increases at later stages of the disease. Moreover, significant differences were evident in several AS comparisons. Specifically, there were significant differences for all CN vs. CDR $\ge$ 0, and CDR = 0 vs. CDR = 0.5 adjacent AD dataset pairs. Regarding ADS (see Fig. 9b), it is worth noting that the hippocampus and amygdala regions exhibit the smallest p-values denoting the most statistical significance among the cohorts, which aligns with previous studies. The ADS shows significant differences in the hippocampus and amygdala and no significant differences in ventricle-related regions within AD subgroups. Notably, when examining disease progression, the ADS can provide supplementary information for distinguishing AD progression, as it reveals a significant difference between the CDR = 0.5 vs. CDR = 1 adjacent AD pairs, which may not be readily discernible when considering only AS. It is important to note that the observation for CDR = 2 is based on the limited availability of only four scans in the dataset. Thus, statistical testing was not performed for the CDR = 2 group.

Furthermore, in addition to performing independent t-tests, we reported Cohen’s d effect sizes for AS and ADS. A conventional interpretation of effect sizes categorizes them as small (d = 0.2), medium (d = 0.5), and large (d = 0.8), as suggested in^37^. However, it is essential to note that these values should not be rigidly applied and must be considered in the context of specific research^38^. Typically, a Cohen’s d of 0.5 signifies a difference equivalent to half a standard deviation. In light of this, we have further categorized effect sizes into more refined ranges based on Cohen’s d absolute values, ranging from medium to very large (Medium effect size: [0.35, 0.65); large effect size: [0.65, 0.9); very large effect size: > 0.9), and highlighted them with distinct markings in Table 2.

## Discussion

This study presents a framework to independently quantify brain morphological changes due to normal aging and AD from structural MRI scans. By combining deep learning techniques, and diffeomorphic registration, we propose two scores that can potentially be used for describing mechanisms underlying AD-related changes in brain structure. Overall, the proposed framework aims to advance our understanding of the intricate interplay between normal aging and AD progression. The proposed framework encompasses several steps that collectively contribute to our understanding of this complex relationship.

### Generation of age-specific templates

Accurately describing the voxel-level changes associated with normal brain aging is a complex task, necessitating the consideration of both spatial (inter-subject variation) and temporal (intra-subject registration) dynamics. Many neuroimaging studies use standard templates (e.g., the classical MNI-152 template), an average image of the dataset, or one specific image from the dataset to bring all images into the same space before further analyses. One of the issues of the former approaches is that these are relatively blurred, compromising the accuracy of registrations. In turn, a specific subject might not be at the barycenter of the dataset, leading to biases. Also, these strategies suffer from not being age-specific.

For this reason, we use a DL-based method to generate sharp, age-specific templates representing the CN subjects in the OASIS-3 dataset. In particular, we trained the neural network proposed by^17^ with our data. As sharpness is desirable to increase the accuracy of downstream processing (e.g. segmentation and registration), we test different hyperparameters to minimize the EFC (sharper images have lower EFC).

It is worth noting that the pursuit of sharper images for the registration algorithm introduces certain limitations, as discussed in^13^. Specifically, a high-intensity gradient along boundaries can lead to larger deformations outside the target organ, in our case, the brain. This phenomenon might significantly impact deformation-dependent measurements. Fortunately,^13^ has shown the efficacy of introducing masks to mitigate the influence of background artifacts. In our study, we also address this by introducing the segmentation mask, which helps counteract the impact of background errors.

As discussed in^31^, anatomical plausibility is usually an issue for DL-based image generation. To target this issue, state-of-the-art DL-based diffeomorphic registration methods integrate a non-DL-based module to integrate the SVFs^39^. This way, diffeomorphism can be guaranteed and, hence, the anatomical plausibility of the templates. Moreover, the diffeomorphic registration module in the framework ensures that the learned templates accurately represent training samples proximal to the target age while preserving inter-subject topology. The continuous age condition further contributes to the templates’ coherent evolution. We found that the asymmetry of both the average and learned templates increases with age. This is inline with recent large-cohort studies^40,41^ that have shown that asymmetries in gray and white matter metrics increase with age, particularly later in life.

Another challenge of DL-based methods is that their performance usually decreases beyond the scope of training data. Notably, current AD-targeted datasets are inherently skewed toward individuals at high risk of developing AD, resulting in an imbalanced age distribution within the data (refer to Fig. 2). To mitigate potential model bias toward the majority age group, we employ a strategy during training to get a similar representation of images at all ages during training. The outcomes illustrated in Fig. 4 show that the learned templates accurately capture underlying trends in different brain regions. As shown in that figure, the trend of the templates is approximately in the middle of the curve of inter-subject variability of the CN dataset. Moreover, it can be noticed that the CN cohort has a lower inter-subject variability compared with the AD cohort.

Besides the imbalanced age distribution problem, we previously examined in^31^ how domain shifts from training data to unseen data can damage registration performance, thereby requiring compensation to take place by introducing a stopping point different from one in the integration layer. Since the training and testing images in this study come from the same dataset, this adjustment is not required. Still, this is an issue to consider when multiple datasets are used.

### Aging and AD-specific scores

Studies have shown the intertwined relationship between aging and AD, suggesting that AD could partly reflect an accelerated brain aging process^42–44^. Moreover, it has been observed that normal aging manifests differently in different brain regions^42^, and certain structures such as the hippocampus and amygdala are more susceptible to AD^45–47^. However, precisely describing normal aging, particularly involving localized brain aging is complex. Furthermore, disentangling normal aging components from disease-specific changes poses additional challenges. Utilizing a refined score for normal aging at each voxel offers a means to observe disease-specific changes locally accurately.

We propose AS and ADS as features that could potentially be used as imaging biomarkers in AD. For this, we first define a unit-year normal aging SVF using DL-based diffeomorphic registration between the generated templates of CN subjects at 60 and 90 years old. This SVF captures the intricate transformations between templates, thereby identifying voxel-level anatomical changes associated with normal aging. In the next step, we register the given image to the 60-year-old template and perform a voxel-wise projection between the obtained SVF and the reference unit-year normal aging SVF. The parallel and orthogonal components of the projection are used to estimate AS and ADS, respectively. In order to increase the robustness of the estimations, voxels with too small unit-year normal aging are removed. It is important to note that our method follows the assumption that normal aging evolves linearly over time, based on the average group-wise SVF $v_0$, as described in previous work^2^. While the learning-based approach employed in this study enables the generation of age-specific templates, which could be used to test non-linear aging progressions for potentially greater accuracy, we adhered to the linear assumption for this study. This decision aligns with prior evidence supporting the linearity of normal aging, particularly in older populations^2,29,32,33^. Moreover, as shown in Fig. 4, both the generated templates and the underlying real data exhibit approximately linear volumetric changes with age, providing further validation for this assumption.

### Evaluation on the CN cohort

The performance of AS and ADS is evaluated using 326 MRI scans, all of which have a consistent CDR score of 0 across longitudinal MRI sessions. Many studies have highlighted the fact that brain aging occurs at varying rates across different brain regions^32,44,48^. While ventricle enlargement is a common observation in both the normal aging process and AD progression^49,50^, AD is characterized by more pronounced atrophy in regions such as the hippocampi and amygdala^51–53^. Employing segmentation allows us to perform regional analyses. In particular, we investigate ventricles, hippocampi, and amygdala regions in addition to the whole brain. By aligning different age cohorts to a common anatomy, such as the template of 60 years-old in our study, we enable meaningful inter-group structural comparisons.

As mentioned, we used a thresholding strategy to filter outliers based on the magnitude of $\varvec{v}_0$ for the same anatomy. We examined the correlation between chronological age and the AS at different thresholding quantiles. We used $R^2$ to determine the best threshold per region as shown in Fig. 5. Considering an assumption that morphological age corresponds to chronological age, the AS should precisely reflect the normal aging process, exhibiting linear changes with age as shown in Fig. 6a (assuming the reference template corresponds to the age of 60). However, morphological changes do not always exhibit a simple equivalent correlation with increasing age. We find that different regions exhibit distinct optimal quantile thresholds for outlier removal. This variability might arise from factors such as considerable inter-subject anatomical variation.

Many neurodegeneration studies have highlighted the considerable inter-subject variation even within cognitively normal populations^32,48,54–56^. This inherent variability also affects the estimations of the AS, which might contribute to the relatively low $R^2$ scores in Fig. 6a. Although the $R^2$ scores for AS are relatively low, the p-values emphasize the significant correlation between chronological age and AS scores in the observed MRI regions. Conversely, despite the very small $R^2$ scores for ADS, indicating a weak correlation between chronological age and ADS scores, the p-values underscore the importance of accounting for age effects for certain MRI regions. Consequently, we employed a normalization procedure when comparing groups with substantial age distribution shifts.

Two notable sources of error deserve discussion. Firstly, the registration algorithm yields larger deformations for inter-subject registrations compared to intra-subject ones. The intricate nature of brain sulci and giri, which might substantially vary among individuals, is analogous to a brain print similar to a fingerprint^57^. This makes it less evident the decomposition of the SVFs into their normal aging and AD-specific components in cortical regions. Secondly, we identified a potential registration error in regions that exhibit homogeneous intensity, such as the ventricles. In these cases, deformation vectors could become minute. For this reason, we perform regional analyses on the region close to the edges of the ventricles where registration is more reliable, as shown in the fourth column in Figs. 6 and 9.

### Evaluation on the AD cohort

Subsequently, the performance of AS and ADS are assessed in the AD cohort using the CN group as a comparison. The AD cohort comprised 688 MRI scans with varying CDR scores ranging from cognitively normal (CDR = 0, notice that while CDR = 0 is a marker of normal cognition, these subjects developed AD later in time. For this reason, they are part of the AD cohort and are excluded from the CN cohort) to moderate AD dementia (CDR = 2). It is worth mentioning that OASIS-3 focused on the enrollment of early to mild AD individuals. That is why the CDR > 2 stages are not represented in the dataset. The terms “early stage” and “later stage” of AD were used in our discussion to indicate the relative stage in the range of early-stage AD dementia of the dataset, which is more difficult to differentiate. Results are presented in boxplots along with p-value levels on each subfigure of Fig. 9, as well as pair-wise Cohen’s d estimates in Table 2 to quantify the effect sizes of the differences.

When examining the ability of the two scores to track disease progression, we found that the two scores are indispensable and can be complementary. For example, by inspecting the boxplots, we found obvious ascending trends with an increase of disease stages in AD subgroups in all four observed brain regions for AS as illustrated in Fig. 9a, and in hippocampi & amygdala regions for ADS in Fig. 9b, despite the absence of a notable distinction across AD subgroups in chronological age distributions, as shown in Fig. 8. In addition to the ascending trends over the whole AD course, the two scores exhibit different sensitivities in different stages of AD progression by examining the p-values and effect sizes. The ADS seems to have higher sensitivity for the later stage of AD. For example, there is no statistical difference by using AS in any MRI region for a later stage between CDR 0.5 vs CDR 1 pair until we consider the hippocampi & amygdala regions by using ADS for this pair. The bigger effect sizes for ADS compared with AS (i.e., -0.37 vs. -0.06 in the hippocampi & amygdala regions, -0.20 vs. -0.07 in the whole brain region in Table 2) in this pair further validates that. In contrast, AS has a better performance than ADS in the early stage, such as CDR 0 vs. CDR 0.5. The bigger effect sizes can be observed in all four MRI regions for AS compared with ADS (i.e., (0, 0.5) column in Table 2) in this pair. An intriguing observation is the noticeable differences between the CN and CDR = 0 AD subgroup by using both scores. This might suggest that morphological changes manifest prior to detectable cognitive alterations in clinical instruments such as the CDR.

The coupling of the results from the two scores suggests that ventricles predominantly follow accelerated normal aging in AD, while the atrophy in hippocampi & amygdala regions is influenced by both normal aging and AD-specific directions. Table 2 shows that disentangling normal aging from AD-specific factors can reveal a transferred atrophy pattern in hippocampi & amygdala regions. For example, in the pair (CN, 0), the effect sizes of both scores are very similar (i.e., 0.22 vs. 0.24), while in the early stage in AD, AS contributes more than ADS (i.e., 0.41 vs. 0.32 in pair (0, 0.5)), then in the later stage, ADS dominates more than AS (i.e., 0.06 vs. 0.37 in pair (0.5, 1)).

To summarize, the experiments on the OASIS-3 dataset using the two introduced scores revealed different atrophy patterns in terms of AD progression and regions affected by AD. Specifically, we show that the enlargement of ventricles in AD is contributed predominantly by accelerated normal aging direction and is less associated with morphological changes caused by AD-specific direction. In contrast, hippocampi & amygdala regions are influenced more by normal aging direction at early AD stages and then transferred to be influenced more by AD-specific direction at late AD stages.

### Relationship with classical morphometric studies

Voxel-based morphometry (VBM) and tensor-based morphometry (TBM) methods have been widely used in group analyses between cognitively healthy and AD groups^58–60^. These methods usually employ scalars, such as the Jacobian determinant, to illustrate local expansion and contraction patterns in gray matter^61^. While our proposed method also uses registration, the goal is to assess whether the detected changes in the brain follow the patterns of normal aging or not.

Our study adopts a deformation-based morphometric approach, enabling the isolation of the normal aging and AD-specific components-attributes not attainable through VBM or TBM. Central to conventional morphometric group-wise studies is the construction of a target-specific template. Historically, this process involved resource-intensive registration computations between each pair of intra-subject imaging data, occasionally necessitating the use of mathematically demanding tools like parallel transport to transfer individual trajectories to template space^2,13^. However, in our study, we take advantage of the efficiency of deep learning methods for both template creation and registration steps. By leveraging the power of deep learning, our framework offers several advantages over traditional morphometric approaches. The integration of DL-based diffeomorphic registration not only enhances the accuracy of template creation but also facilitates the extraction of nuanced anatomical patterns associated with normal aging in an efficient manner.

### Limitations and future work

Some limitations should be acknowledged. Firstly, our framework is trained and evaluated solely on the OASIS-3 dataset, which may introduce dataset-specific biases and potentially limit the generalizability of our findings. For example, the creation of normal aging templates is based on 1,352 scans from the CN group within this dataset, which might be biased by the fact that OASIS-3 predominantly included participants in specific geographical regions (i.e., primarily the United States) and exhibits an unbalanced representation of racial diversity (e.g., only 5 Asian participants are reported in the demographics). Consequently, the normal aging patterns derived from this dataset might not be fully representative of the broader human population. So one needs to consider this effect when applying the learned normal aging pattern to datasets from different domains. Although other publicly available datasets, e.g., ADNI, are extensively used for AD research, we decided not to incorporate it in this study to avoid issues associated with domain shifts between different datasets^31^, ensuring more reliable and interpretable results as assessed in the same dataset. Another limitation is that we only used age to create conditional templates. With access to larger datasets, it would be possible to assess other the proposed scores for different genders.

Additionally, it is important to acknowledge that the approach of determining the AD-specific component through the average magnitude of the residual vector is not without limitations. The registration process between a reference CN template and each subject’s scan can be considered an inter-subject scenario. Consequently, the residual vector may contain both AD-specific and subject-specific components. While steps were taken to mitigate the influence of subject-specific differences through affine registration and an outlier rejection strategy, the development of a more sophisticated model that explicitly addresses these factors is a worthwhile pursuit for achieving a more accurate and interpretable model. Some methods have emerged to tackle this inter-subject variability by explicitly modeling aging and disease severity. For instance,^62^ introduced orthogonal constraints within the latent space of a variational autoencoder. However, this approach’s learned global coefficient does not offer regional information. Another approach, as seen in^29^, explicitly models both components along with an inter-subject component through longitudinal data. While the incorporation of longitudinal data offers potential benefits in building a comprehensive and robust model, such data is often limited within a single dataset. Future studies could consider augmenting the datasets with more longitudinal data or including a bigger longitudinal dataset to better address inter-subject differences. It is worth noting that while our approach does not specifically account for subject differences, the results remain valid when comparing across groups under the assumption that group-wise subject-level differences are consistent.

Finally, notice that we assume a linear atrophy due to normal aging in our experiments. Since we can compute templates at every age, it would be possible to estimate an age-dependent reference SVF $\varvec{v}_0$. Indeed, this will require different normalization procedures for estimating AS and ADS. This is part of our current research efforts.

## Conclusion

In summary, our framework can be used to investigate the intricate interplay between AD and normal aging in terms of brain atrophy, addressing critical questions about their relationship. Leveraging deep learning advancements, we construct age-specific templates and delineate normal aging atrophy patterns through advanced registration techniques. The extracted voxel-level vectors reveal nuanced variations attributed to normal aging, fostering a more robust evaluation of their connection to AD progression. Our study uncovers that the impact of AD is twofold: the trajectory of normal aging-related brain atrophy gets faster with AD in certain brain regions (e.g., both ventricles and hippocampi & amygdala), while other regions exhibit distinctive AD-specific atrophy patterns (e.g., hippocampi & amygdala). Combining these components may in the future enable differentiation of subtle AD clinical stages. In essence, our work contributes valuable insights into the convergence of normal aging and AD.

## Data Availability

The data that support the findings of this study are openly available in OASIS at http://doi.org/10.1101/2019.12.13.19014902, reference number^18^. The source codes generated for this study are available on https://github.com/Fjr9516/DBM_with_DL.
